# Determinants of low birth weight babies delivered at paropakar maternity and Women’s hospital: A case-control study

**DOI:** 10.1371/journal.pone.0335107

**Published:** 2026-06-22

**Authors:** Sadiksha Pokhrel, Paras Kumar Pokharel, Anup Ghimire, Birendra Kumar Yadav, Avaniendra Chakravartty, Swastika Poudel, Laxmiswori Prajapati, Akshaya Acharya

**Affiliations:** 1 School of Public Health and Community Medicine, BP Koirala Institute of Health Sciences, Dharan, Nepal; 2 Paropakar Maternity and Women’s Hospital, Kathmandu, Nepal; 3 Central Department of Public Health, Institute of Medicine, Tribhuwan University, Kathmandu, Nepal; University of California San Diego, UNITED STATES OF AMERICA

## Abstract

**Background:**

Low birth weight remains a major public health concern due to its association with adverse neonatal, developmental, and long-term health outcomes, with no substantial improvement observed over the past decade in Nepal. Multiple socioeconomic, maternal, and obstetric factors contribute to low birth weight, particularly in low-resource settings. This study aimed to identify the determinants of low birth weight babies delivered at Paropakar Maternity and Women’s Hospital in Kathmandu, Nepal.

**Methods:**

A hospital-based age-matched (± 5 years) case-control study was carried out from 1^st^ August 2024–30^th^ January 2025 among mothers who delivered live babies at Paropakar Maternity and Women’s Hospital. A total of 57 cases (birth weight < 2500 g) and 114 controls (birth weight ≥2500 g) in the ratio of 1:2 were selected. Data on exposure variables were collected through interviews and medical records review. Chi-square test, Mann-Whitney U test and binary logistic regression were performed at 95% confidence interval (CI) to find the significant determinants of low birth weight by using SPSS version 11.5.

**Results:**

Multivariate logistic regression analysis revealed that independent risk factors of LBW babies were educational status of mother (AOR: 6.32; 95% CI 1.90 to 21.05), per capita income (AOR: 2.89; 95% CI 1.02 to 8.18), parity (AOR: 4.80; 95% CI 1.91 to 12.07), hemoglobin level (AOR: 6.19; 95% CI 1.79 to 21.38), period of gestation (AOR: 8.16; 95% CI 2.42 to 27.49), weight before pregnancy (AOR: 4.86; 95% CI 1.02 to 23.29), history of chronic medical illness (AOR: 8.22; 95% CI 2.25 to 29.99) and illness during pregnancy (AOR: 3.33; 95% CI 1.20 to 9.28), type of diet (AOR: 4.84; 95% CI 1.14 to 20.64).

**Conclusion:**

The findings emphasize the importance of maternal health, nutrition, and prenatal care in preventing low birth weight. Interventions targeting maternal education, nutritional status, regular antenatal care, and timely management of maternal illnesses may reduce the incidence of low birth weight.

## Introduction

WHO had defined low birth weight as the weight of infants less than 2500 grams at birth irrespective of the gestational age. [1] The proportion of babies with LBW is considered a sensitive index that represents the country’s health and development. [2] The babies with LBW are at a greater risk of malnutrition as well as other childhood morbidities such as pneumonia and diarrhea, which are considered the major challenging public health problem. [3] LBW infants lack the strength compared to babies born with normal weight due to their tiny bodies and are at a higher risk to various health-related problems. As they lack body fat, they typically struggle to stay warm and might suffer from hypothermia. The baby’s risk for complications increases with low birth weight. [4–6]

LBW babies are more likely to die during the first month of their life. They might face lifelong consequences including a higher risk of stunted growth, lower IQ, and adult-onset chronicconditions such as hypertension, ischemic heart disease, stroke, metabolic syndrome, obesity, diabetes, malignancies, osteoarthritis and dementia. [7,8] It is also associated with an increased risk of behavioral problems, psychological illnesses, learning difficulties, and sensory impairments that affect cognitive function in developing children and adolescents that provide significant challenges to the individual’s educational and quality of life outcomes. [2]

It has been estimated that 15–20% of infants worldwide weigh less than2500 grams at birth, which is equal to more than 20 million births annually. [9] Worldwide, one out of seven newborns is affected due to LBW. [7] Out of total LBW babies worldwide, 95% occur in developing nations, which is more than twice as high as in developed nations, i.e., 16.5% and 7.0% respectively.[2,10] Southern Asia (27%) has the highest incidence of LBW followed by Africa (14%), Latin America and the Caribbean (9%), and Eastern Asia (6%). [8,11,12] Based on the data from the Global Nutrition Report, 12.3% of newborns in the South-eastern Asia sub-region have low birth weight. [13]

In Nepal, the percentage of newborns with low birth weight has slightly decreased from 12.8% in FY 2076/77 to 11.2% in FY 2077/78. According to the Nepal Demographic Health Survey (NDHS) 2016, the prevalence of low birth weight is 12%. Likewise, the data from the NDHS 2022 reported that 5% of babies were very small at birth and 10% were smaller than average. Based on these data, during the past decade, the proportion of newborns with low birth weight has remained stagnant. [14,15] LBW is influenced by a complex interplay of socio-demographic and environmental factors, including poverty, maternal smoking, and inadequate access to healthcare. Understanding the role of these factors in the context of Nepal is essential for designing effective interventions. This research has the potential to improve maternal and child health outcomes, reduce neonatal mortality, and contribute to achieving Sustainable Development Goal 3 while addressing the unique challenges and regional variations within Nepal by identifying the modifiable risk factors that are associated with the delivery of low birth weight babies.

Despite several studies on low birth weight (LBW) in Nepal, evidence from Kathmandu Valley is limited. Paropakar Maternity and Women’s Hospital, the country’s largest referral center, serves mothers from both urban and rural backgrounds, offering a unique setting to study diverse risk factors. With LBW rates in Nepal showing little improvement in the past decade (NDHS 2022), updated hospital-based evidence from this context is essential to guide effective interventions. Thus, this study aimed to identify the risk factors associated with low birth weight babies delivered at Paropakar Maternity and Women’s Hospital in Kathmandu, Nepal.

## Materials and Methods

### Study design and setting

A hospital-based age-matched case-control design (age of mothers ± 5 years) was employed in this study to identify the determinants of low birth weight babies. Paropakar Maternity and Women’s Hospital, Kathmandu, was purposively selected as the study setting. Data collection was done from 1^st^ August 2024–30^th^ January 2025.

### Participants

**Cases:** Women delivering newborns with a birth weight less than 2500 grams at Paropakar Maternity and Women’s Hospital.

**Controls:** Women delivering newborns with birth weight greater than or equal to 2500 grams at Paropakar Maternity and Women’s Hospital.

### Inclusion and exclusion criteria

All women who delivered singleton live babies at the hospital during the data collection period were included in the study, while those women who gave birth to twin babies were excluded.

### Sample size and sampling technique

The number of cases and controls was calculated by using OpenEpi, Version 3 by taking; power at 80%, a two-sided significance level at 0.05, percentage of controls exposed 35.41, and odds ratio of 2.51 by taking gestational age as an exposure variable based on the study conducted by Shrestha et al. (2020) at Lumbini Provincial Hospital. [16] The ratio of cases to controls was taken as 1:2. Thus, the required sample size calculated was 57 cases and 114 controls.

The delivery record book was checked each day during the data collection period in the Post Natal Care ward of Paropakar Maternity and Women’s Hospital for the delivery of low birth weight babies. Mothers who gave birth to LBW babies (birth weight < 2500g) were taken as cases for the data collection. Similarly, two controls (birth weight ≥ 2500g) were selected on the same day when a case was found for the data collection by matching the age with the case.

### Validity and reliability

To ensure validity and reliability, the questionnaire to assess the risk factors of LBW babies was prepared from an extensive literature review. The prepared structured questionnaire was translated into the Nepali language and then back-translated into English to ensure its validity and reliability. Pre-testing of the questionnaire was done on 10% of study the sample in a similar setting which were not included in the main study and required changes were made accordingly.

### Data collection tools and techniques

The pre-tested structured standard questionnaire was used to conduct the study. Face-to-face interviews using the structured questionnaire were conducted for data collection. Some medical and obstetric information obtained from the participants was cross-verified by reviewing their medical records.

### Ethics approval and consent to participate

Ethical approval was obtained from the Institutional Review Committee (IRC), BP Koirala Institute of Health Sciences, Dharan (Reference number: IRC/30/081/82). Permission to conduct the study was also obtained from Paropakar Maternity and Women’s Hospital, Kathmandu. Written informed consent was obtained from all participants before data collection. Confidentiality and anonymity of the respondents were maintained throughout the study by using unique participant codes instead of personal identifiers.

### Variables

Independent variables: Socio-demographic characteristics, Maternal and obstetric factors, and Nutritional and behavioral factors of mothers.

Outcome variable: Low birth weight baby as defined by birth weight < 2500 grams.

### Statistical analysis

Data were collected in paper-based questionnaires and entered into Microsoft Excel 2010, which was then exported to Statistical Package for Social Sciences (SPSS) 11.5 version for statistical analysis. Descriptive and inferential statistical methods were used to analyze and compare various characteristics among mothers of case and control groups, and to identify associations between various factors and delivery of low birth weight babies. The Kolmogorov – Smirnov test and Shapiro – Wilk test, Q-Q plot, Skewness and Kurtosis, Histogram and Box and Whisker plot were used to evaluate whether the data were normally distributed.

For the descriptive analysis, categorical data of both cases and controls were summarized in terms of frequency and percentage. The continuous variables were presented as Median and Inter-Quartile Range (IQR).

For the inferential statistics, bivariate analysis was done using the chi-square test and Mann- Whitney U test to determine factors associated with LBW babies at 95% confidence interval (CI) and p-value less than 0.05. Variables with a p-value less than 0.2 in bivariate analysis were entered into the multivariable logistic regression model to identify independent predictors of low birth weight. Binary logistic regression assumptions were assessed and found to be satisfactory, and model estimates were generated using standard maximum likelihood estimation methods.

### Operational definitions

**Per capita income:** Categorized as below or above the poverty line based on the Nepal Living Standards Survey IV (2022–23), which defined the poverty line as NRs. 72,908 per person per year. [17]

**MUAC:** It is categorized as < 23 cm and ≥ 23 cm based on Food and Nutrition Technical Assistance (FANTA). [18]

**Dietary diversity:** It is categorized as an adequate diverse diet and an inadequate diverse diet. Women consuming 5 of 10 food groups in the past 24 hours while they were pregnant are defined as consuming a diverse diet using the Minimum Dietary Diversity Score for Women (MDD-W) tool given by FAO (Food and Agriculture Organization). [19]

**Chronic medical illness:** Chronic medical illness is defined as a pre-existing medical illness of the mother with an onset before the current pregnancy.

**Illness during pregnancy:** Illness during current pregnancy is defined as a medical condition that developed during current pregnancy for which medical attention or treatment was sought.

**Vegetarian diet** For this study, a vegetarian diet is defined as a dietary pattern in which the mother consumed only plant-based foods such as cereals, pulses, legumes, vegetables and fruits along with dairy products, while excluding all types of meat, poultry, fish and eggs.

## Results

### Socio-demographic characteristics of respondents

Table 1 presents the socio-demographic characteristics of the respondents. More than half of the newborns were male (53.2%). About one-third of the respondents were Janajati (33.3%), more than two-thirds of the respondents followed the Hindu religion (69.6%) followed by Buddhism (15.2%). About 13% of the mothers were illiterate and most of the mothers were homemakers (69.6%). More than half of the respondents belonged to nuclear families (51.5%) and 76.6% of the respondents lived in a family with fewer than five members. The median monthly family income of the respondents was NRs 36,000 and about one-fourth of the participants were below the poverty line (23.98%).

**Table 1:** Socio demographic characteristics of respondents.

Variables	Case (57)	Control (114)	Total (171)
	n (%)	n (%)	n (%)
**Sex of child**			
Female	30 (53.6%)	50 (43.9%)	80 (46.8%)
Male	27 (47.4%)	64 (56.1%)	91 (53.2%)
**Ethnicity**			
Brahmin	17 (29.8%)	17 (14.9%)	34 (19.9%)
Chhetri	9 (15.8%)	19 (16.7%)	28 (16.4%)
Newar	7 (12.3%)	10 (8.8%)	17 (9.9%)
Dalit	6 (10.5%)	16 (14.0%)	22 (12.9%)
Janajati	15 (26.3%)	42 (36.8%)	57 (33.3%)
Others (Muslim, Madhesi)	3 (5.3%)	10 (8.8%)	13 (7.6%)
**Religion**			
Hindu	41 (71.9%)	78 (68.4%)	119 (69.6%)
Buddhist	10 (17.5%)	16 (14%)	26 (15.2%)
Muslim	2 (3.5%)	5 (4.4%)	7 (4.1%)
Kirat	1 (1.8%)	8 (7%)	9 (5.3%)
Christian	3 (5.3%)	7 (6.1%)	10 (5.8%)
**Educational status of the mother**			
Illiterate	12 (21.1%)	10 (8.8%)	22 (12.9%)
Literate	45 (78.9%)	104 (91.2%)	149 (87.1%)
**Occupation of the mother**			
Agriculture	2 (3.5%)	6 (5.3%)	8 (4.7%)
Government Job	1 (1.8%)	3 (2.6%)	4 (2.3%)
Business	5 (8.8%)	8 (7.0%)	13 (7.6%)
Home maker	40 (70.2%)	79 (69.3%)	119 (69.6%)
Private Job	7 (12.3%)	13 (11.4%)	20 (11.7%)
Daily wage	2 (3.5%)	5 (4.4%)	7 (4.1%)
**Family size**			
≥ 5 members	16 (28.1%)	24 (21.1%)	40 (23.4%)
< 5 members	41 (71.9%)	90 (78.9%)	131 (76.6%)
**Monthly income (in NRs)**			
Median (IQR)	35000 (25000 - 50000)	40000 (28750 - 50000)	36000 (25000 - 50000)
**Per capita income**			
Below Poverty Line	20 (35.1%)	21 (18.4%)	41 (24%)
Above Poverty Line	37 (64.9%)	93 (81.6%)	130 (76%)

### Maternal and obstetric factors of respondents

Out of 171 respondents, more than half of the respondents had a normal delivery (56.1%) and were primiparous (52.6%). The majority of the respondents had an inter-pregnancy interval of more than or equal to two years (88.0%) and attended ANC visits four or more times (95.9%). About 13% of the respondents had hemoglobin level ≥ 11 mg/dl. The gestation period was less than 37 weeks among 14.6% of the respondents. More than one-fourth of the respondents had previously given birth to low birth weight babies (26.3%), 11.7% had an abortion and 8.8% had a history of preterm birth. About 11.0% of the respondents had a height less than or equal to 145 cm and weight less than 45 kg. A history of chronic medical illness was present in 11.1% of the women and 22.2% women had faced illness during pregnancy. (Table 2)

**Table 2:** Maternal and obstetric factors of respondents.

Variables	Case (57)	Control (114)	Total (171)
	n (%)	n (%)	n (%)
**Parity**			
Primiparous	38 (66.7%)	52 (45.6%)	90 (52.6%)
Multiparous	19 (33.3%)	62 (54.4%)	81 (47.4%)
**Inter-pregnancy interval**			
< 2 yrs	1 (5.3%)	9 (14.1%)	10 (12.0%)
≥ 2 yrs	18 (94.7%)	55 (85.9%)	73 (88.0%)
**Frequency of ANC visit**			
< 4 visits	5 (8.8%)	2 (1.8%)	7 (4.1%)
≥ 4 visits	52 (91.2%)	112 (98.2%)	164 (95.9%)
**Hemoglobin level**			
< 11 mg/dl	14 (24.6%)	8 (7.0%)	22 (12.9%)
≥ 11 mg/dl	43 (75.4%)	106 (93.0%)	149 (87.1%)
**Period of gestation**			
< 37 weeks	18 (31.6%)	7 (6.1%)	25 (14.6%)
≥ 37 weeks	39 (68.4%)	107 (93.9%)	146 (85.4%)
**History of LBW**			
Yes	8 (47.1%)	13 (20.6%)	21 (26.3%)
No	9 (52.9%)	50 (79.4%)	59 (73.8%)
**Height**			
≤ 145 cm	7 (12.3%)	12 (10.5%)	19 (11.1%)
> 145 cm	50 (87.7%)	102 (89.5%)	152 (88.9%)
**Weight**			
< 45 kg	13 (22.8%)	6 (5.3%)	19 (11.1%)
≥ 45 kg	44 (77.2%)	108 (94.7%)	152 (88.9%)
**MUAC**			
< 23 cm	15 (26.3%)	5 (4.4%)	20 (11.7%)
≥ 23 cm	42 (73.7%)	109 (95.6%)	151 (88.3%)
**History of chronic medical illness**			
Yes	12 (21.1%)	7 (6.1%)	19 (11.1%)
No	45 (78.9%)	107 (93.9%)	152 (88.9%)
**Illness during pregnancy**			
Yes	17 (29.8%)	21 (18.4%)	38 (22.2%)
No	40 (70.2%)	93 (81.6%)	133 (77.8%)

### Nutritional and behavioral factors of respondents

Among 171 respondents, a significant portion of the respondents (34.5%) did not take any additional meals during pregnancy. The majority of the respondents were non-vegetarian (88.9%) and had taken Iron Folic Acid (IFA) for three months or more during pregnancy. Only 3.5% and 2.9% of the mothers reported smoking and consuming alcohol respectively during pregnancy. Regarding the duration of sleep at night, 30.4% of the participants reported sleeping for less than 8 hours during pregnancy. (Table 3)

**Table 3:** Nutritional and behavioral factors of respondents.

Variables	Case (57)	Control (114)	Total (171)
	n (%)	n (%)	n (%)
**Additional meal during pregnancy**			
No	24 (42.1%)	35 (30.7%)	59 (34.5%)
Yes	33 (57.9%)	79 (69.3%)	112 (65.5%)
**Type of diet**			
Vegetarian	10 (17.5%)	9 (7.9%)	19 (11.1%)
Non-vegetarian	47 (82.5%)	105 (92.1%)	152 (88.9%)
**IFA during pregnancy**			
< 3 months	6 (10.5%)	8 (7.0%)	14 (8.2%)
≥ 3 months	51 (89.5%)	106 (93.0%)	157 (91.8%)
**Dietary diversity**			
Inadequate (< 5)	12 (21.1%)	5 (4.4%)	17 (9.9%)
Adequate (≥ 5)	45 (78.9%)	109 (95.6%)	154 (90.1%)
**Smoking habit**			
Yes	5 (8.8%)	1 (0.9%)	6 (3.5%)
No	52 (91.2%)	113 (99.1%)	165 (96.5%)
**Alcohol consumption**			
Yes	4 (7.0%)	1 (0.9%)	5 (2.9%)
No	53 (93.0%)	113 (99.1%)	166 (97.1%)
**Duration of sleep at night**			
< 8 hours	23 (40.4%)	29 (25.4%)	52 (30.4%)
≥ 8 hours	34 (59.6%)	85 (74.6%)	119 (69.6%)

### Determinants of low birth weight

Bivariate and multivariate logistic regression analyses were performed to determine the association between various risk factors and the birth of LBW babies. The bivariate analyses revealed that the educational status of the mother, per capita income, parity, frequency of ANC visits, hemoglobin level, period of gestation, history of LBW, pre-pregnancy weight, MUAC, history of chronic medical illness, smoking status, alcohol consumption and duration of sleep at night were significantly associated with LBW. Variables that were significantly associated with LBW in bivariate analyses and with a p-value less than or equal to 0.2 were subjected to multivariate analysis using the enter method and the variables having expected cell count less than five were excluded. Table 4 shows that illiterate women were six times more likely to give birth to LBW babies as compared to literate women (AOR: 6.32; 95% CI 1.90 to 21.05). Similarly, women whose per capita income was below the poverty line had almost three times higher risk of giving birth to LBW babies (AOR: 2.89; 95% CI 1.02 to 8.18).

**Table 4:** Bivariate and multivariate logistic regression analysis of socio-demographic factors associated with low birth weight babies.

Variables	p-value	Crude OR (95% CI)	p-value	Adjusted OR (95% CI)
**Sex of child**				
Female	0.278^**a**^	1.42 (0.75–2.69)		
Male	Reference			
**Ethnicity**				
Others*	0.072^**a**^	0.55 (0.29–1.06)	0.388	0.67 (0.27–1.66)
Brahmin/Chhetri	Reference		Reference	
**Religion**				
Non-hindu	0.638^**a**^	0.85 (0.42–1.70)		
Hindu	Reference			
**Educational status of the mother**				
Illiterate	**0.024** ^**a**^	**2.77 (1.12–6.88)**	**0.003**	**6.32 (1.90–21.05)**
Literate	Reference		Reference	
**Occupation of the mother**				
Home maker	0.906^**a**^	1.04 (0.52–2.08)		
Employed	Reference			
**Family size**				
≥ 5 members	0.307^**a**^	1.46 (0.70–3.04)		
< 5 members	Reference			
**Per capita income**				
Below Poverty Line	**0.016** ^**a**^	**2.39 (1.16–4.92)**	0.046*	**2.89 (1.02–8.18)**
Above Poverty Line	Reference		Reference	

^a^ = Pearson chi- square; ^b^ = Fischer’s exact test, *Significant at p-value < 0.05.

Table 5 shows that primiparous women were almost five times more likely to give birth to LBW babies compared to multiparous women (AOR: 4.80; 95% CI 1.91 to 12.07). Anemic women had about six times higher odds of giving birth to LBW babies (AOR: 6.19; 95% CI 1.79 to 21.38). Preterm babies were eight times more likely to be low birth weight as compared to term infants (AOR: 8.16; 95% CI 2.42 to 27.49). Those women whose weight was less than 45 kg before pregnancy had almost five times increased odds of giving birth to LBW babies (AOR: 4.86; 95% CI 1.02 to 23.29). Women with a history of chronic medical illness were eight times more likely to have LBW babies (AOR: 8.22; 95% CI 2.25 to 29.99). Women who had experienced illness during pregnancy were at three times increased odds of having LBW babies as compared to those who had not experienced any illness during pregnancy (AOR: 3.33; 95% CI 1.20 to 9.28).

**Table 5:** Bivariate and multivariate logistic regression analysis of mateenal and obstetric factors associated with low birth weight babies.

Variables	p-value	Crude OR (95% CI)	p-value	Adjusted OR (95% CI)
**Parity**				
Primiparous	**0.009** ^**a**^	**2.39 (1.13–4.63)**	0.001*	**4.80 (1.91–12.07)**
Multiparous	Reference		Reference	
**Inter-pregnancy interval**				
< 2 years	0.441^b^	0.34 (0.04–2.87)		
≥ 2 years	Reference			
**Frequency of ANC visit**				
< 4 visits	**0.042** ^**b**^	**5.39 (1.01–28.68)**		
≥ 4 visits	Reference			
**Hemoglobin level**				
< 11 mg/dl	**0.001** ^**a**^	**4.31 (1.69–11.02)**	0.004*	**6.19 (1.79–21.38)**
≥ 11 mg/dl	Reference			
**Period of gestation**				
< 37 weeks	**<0.001** ^**a**^	**7.06 (2.74–18.19)**	0.001*	**8.16 (2.42–27.49)**
≥ 37 weeks	Reference			
**History of LBW**				
Yes	**0.028** ^**a**^	**3.42 (1.10–10.59)**		
No	Reference			
**Height**				
≤ 145 cm	0.731^**a**^	1.19 (0.44–3.21)		
> 145 cm	Reference			
**Weight before pregnancy**				
< 45 kgs	**0.001** ^**a**^	**5.32 (1.90–14.88)**	0.048*	**4.86 (1.02–23.29)**
≥ 45 kgs	Reference		Reference	
**MUAC**				
< 23 cm	**<0.001** ^**a**^	**7.79 (2.66–22.76)**	0.722	0.15 (0.15–4.06)
≥ 23 cm	Reference		Reference	
**History of chronic medical illness**				
Yes	**0.003** ^**a**^	**4.08 (1.51–11.03)**	0.001*	**8.22 (2.25–29.99)**
No	Reference		Reference	
**Illness during pregnancy**				
Yes	0.091^**a**^	1.88 (0.89–3.94)	0.021*	**3.33 (1.20–9.28)**
No	Reference		Reference	

^a^ = Pearson chi- square; ^b^ = Fischer’s exact test, *Significant at p-value < 0.05.

Table 6 shows that vegetarian mothers were almost five times more likely to give birth to LBW babies as compared to non-vegetarian mothers (AOR: 4.84; 95% CI 1.14 to 20.64).

**Table 6:** Bivariate and multivariate logistic regression analysis of nutritional and behavioral factors associated with low birth weight babies.

Variables	p-value	Crude OR (95% CI)	p-value	Adjusted OR (95% CI)
**Additional meal during pregnancy**				
No	0.139^**a**^	1.64 (0.85–3.17)	0.105	2.05 (0.86–4.90)
Yes	Reference		Reference	
**Type of diet**				
Vegetarian	0.058^**a**^	2.48 (0.95–6.51)	0.033*	**4.84 (1.14–20.64)**
Non-vegetarian	Reference		Reference	
**IFA during pregnancy**				
< 3 months	0.555^**b**^	1.56 (0.51–4.73)		
≥ 3 months	Reference			
**Smoking habit**				
Yes	**0.016** ^**b**^	**10.86 (1.24–95.35)**		
No	Reference			
**Alcohol consumption**				
Yes	**0.043** ^**b**^	**8.53 (0.93–78.17)**		
No	Reference			
**Duration of sleep at night**				
< 8 hours	**0.046** ^**a**^	**1.98 (1.01–3.90)**	0.842	1.10 (0.44–2.77)
≥ 8 hours	Reference		Reference	

^a^ = Pearson chi- square; ^b^ = Fischer’s exact test, *Significant at p-value < 0.05.

## Discussion

This study analyzed the socio-demographic factors, maternal and obstetric factors and behavioral factors of mothers associated with the delivery of a low birth weight baby. Our study found that maternal educational status was significantly associated with low birth weight, with illiterate mothers more likely to deliver LBW babies than literate mothers. This finding is in line with the studies conducted in Nepal, Ethiopia and Ghana which also reported higher LBW risk among mothers with lower educational attainment. [16,20,21] The observed association may be explained by the influence of education on health literacy, healthcare utilization, nutritional practices, and decision making during pregnancy. Educated mothers are more likely to recognize the importance of antenatal care, adopt healthy behaviors and seek timely medical attention, all of which contribute to improved pregnancy outcomes. However, no statistically significant association was observed between the mother’s educational status and LBW baby in the study conducted in Bangladesh, possibly due to the relative homogeneity of educational levels in the study population, which might have limited detectable differences. [22]

In addition to the educational status, socioeconomic conditions also influenced birth outcomes. A significant association between family per capita income and low birth weight (LBW) was observed where mothers from families below the poverty line had a higher likelihood of delivering LBW infants compared to those from families above the poverty line. Similar findings were observed in a study conducted by Bhaskar et al. that revealed a significant association between per-capita income and LBW. [23] Economic status is found to be a relatively well-established factor for LBW babies as it is linked with overall care for the mothers during pregnancy, which directly impacts the birth outcome. Beyond socioeconomic characteristics, the present study also identified parity as a significant determinant ofLBW, with primiparous women being at a five-fold increased risk of giving birth to LBW babies compared to multiparous women. This finding is consistent with most studies conducted in Nepal, Ghana and several other settings. [12,24,25] The increased risk among first-time mothers (primiparous women) may be related to factors such as lack of experience in prenatal care, poorer nutritional status, or anxiety during pregnancy. Although some studies conducted in Nepal and India have reported no significant association. [26,27]

Additionally, mothers with hemoglobin levels < 11 mg/dl had more than six times the increased risk of giving birth to LBW babies compared to those with hemoglobin levels ≥ 11 mg/dl in this study. This finding aligns with evidence from Ethiopia [28] and other studies by Kayastha et al., Sah et al., Bater et al. and Arabzadeh et al., which also reported a significant association between low maternal hemoglobin levels and the incidence of LBW babies. [12,29–31] However, there are studies, such as those conducted in Lumbini Provincial Hospital in Eastern Nepal, that contradict these findings, as they observed no significant association between maternal hemoglobin levels and LBW babies. [16,23] Physiologically reduced hemoglobin levels hinder the mother’s capacity to deliver adequate oxygen and nutrients to the fetus, contributing to fetal hypoxia and impaired growth, ultimately increasing the risk of LBW. [32]

In addition to maternal hemoglobin status, pregnancy-related factors played an important role in determining birth weight outcomes. Preterm birth is widely recognized as a significant risk factor forLBW, as babies born before 37 weeks of gestation are often not fully developed, leading to lower birth weights. In our study, women who delivered preterm were found to be 8 times more likely to have an LBW baby compared to those who carried their pregnancies to full term. This finding from our study is comparable to those reported in several other studies by Chhetri et al. and Sutan et al. which have also identified preterm delivery as a significant predictor of LBW. [33,34] The strong association is biologically plausible, as preterm infants have less time for intrauterine growth due to which babies born prematurely are more likely to have lower birth weight irrespective of other maternal characteristics. Thus, the consistent findings across multiple studies highlight the importance of preventing preterm births to reduce the incidence of LBW and improve overall neonatal health outcomes.

Beyond pregnancy related factors, maternal nutritional status before conception may also influence fetal growth and birth weight. Our study found that being underweight, with a pre-pregnancy weight below 45 kg, is significantly associated with higher odds of giving birth to a low birth weight baby. This result is consistent with most studies conducted in Ghana, Malaysia, India, Eritrea and Ethiopia [24,34–37] while a few studies by Mulu et al. and Wanode et al. found no significant association between maternal pre-pregnancy weight and the likelihood of delivering a LBW baby. [20,27] This divergence in findings highlights the complexity of maternal health and its influence on neonatal outcomes, underscoring the need for further investigation into the role of pre-pregnancy weight in different demographic and geographic settings.

Apart from the maternal factors, the presence of chronic medical illness among mothers has an increased odds of giving birth to LBW baby in our study which aligns with results from several studies, such as those conducted in India, Nepal, Ethiopia and Malaysia, which identified co-morbid conditions like hypertension as significant risk factors for LBW neonates. [20,34,38,39] This association likely reflects the impact of hypertension, which reduces utero placental blood flow and nutrient delivery to the fetus, ultimately contributing to fetal growth restriction and a higher risk of LBW. [40] Likewise, presence of medical illness during pregnancy was found to be significantly associated with LBW babies in our study which is in accordance with other studies conducted by Bhaskar et al., Toru et al. and Andemariam et al. where the mothers who experienced medical illnesses during pregnancy had a higher risk of delivering LBW infants compared to those who did not face any medical complications during their pregnancy. [23,41,42] Collectively, these findings indicate that maternal illnesses can result in poor pregnancy outcomes by affecting the mother’s overall health, nutritional status, and the ability to carry the pregnancy to full term which increases the likelihood of preterm birth, another significant risk factor for LBW.

The dietary habits of a mother during pregnancy have a significant role in the maintenance of the proper weight of newborn babies. In agreement to this, our study also identified that vegetarian mothers had a higher risk of delivering LBWbabies compared to non-vegetarian mothers. This finding is in line with the study from hospital based study in Nepal and Canada, both of which reported that a plant-based diet was associated with lower birth weight. [29,43] Similarly, another study by Koirala et al. in Nepal also found an increased risk of LBW among vegetarian mothers, though the association was not statistically significant. [44] The association may relate to lower intake of key micronutrients such as iron, vitamin B12 and folate, commonly found in animal-based foods, which are essential for healthy fetal growth. [45] In the Nepalese context, vegetarian diets are commonly practiced due to cultural and religious factors, making this finding particularly relevant. The persistence of this association in the present study highlights the importance of culturally appropriate nutritional counseling and targeted micronutrient supplementation for pregnant women adhering to vegetarian diets.

While dietary practices influence fetal growth through nutrient availability, other maternal behavioral factors may affect birth weight through different biological pathways. Therefore, we also examined the association of smoking and alcohol consumption during pregnancy with LBW. This present study revealed that both smoking and alcohol consumption during pregnancy posed a greater risk of giving birth to LBW babies. These findings are supported by a systematic review and meta-analysis of 55 cohort studies from 1986 to 2020 conducted by Kun Di et al. and an umbrella review conducted by Arabzadeh et al. [31,46] Other studies such as those conducted by Kayastha et al. in Nepal, Taywade et al. in Wardha district of India and Devaguru et al. in Telangana, India, also found results that are consistent with the results of our study. [12,36,47] The association is biologically plausible, as smoking can reduce placental blood flow and oxygen delivery to fetus, while alcohol exposure may impair fetal growth and development. Despite being established risk factors in pregnancy, our study may have lacked sufficient statistical power to detect their effects, as the number of smokers and alcohol consumers was very small.

This study has several limitations. Due to its retrospective design, participants may have had difficulty accurately recalling past exposures, which could have introduced recall bias. The study was conducted in a single government hospital within the Kathmandu Valley so the findings primarily reflect mothers delivering in similar institutional settings and may not capture all community-level variations. Since the data were collected through face-to-face interviews, it might have resulted in social desirability bias in some self-reported behaviors like dietary practices, smoking and alcohol consumption. The relatively small number of participants who reported smoking or alcohol consumption also limited the statistical power to detect strong associations with these factors. Despite these limitations, the findings of this study confirm the continued influence of established socio-demographic, maternal, obstetric and nutritional determinants of low birth weight in the current Nepalese context. Furthermore, the identification of vegetarian dietary practice as a significant predictor highlights the need for culturally appropriate nutritional interventions in populations where vegetarian diets are commonly practiced.

## Conclusions

The study identified key maternal risk factors independently linked to LBW, including the per capita income below the poverty line, higher parity, anemia, preterm delivery (gestational age less than 37 weeks), being underweight before pregnancy, history of chronic medical conditions, illnesses during pregnancy and being vegetarian. These findings underscore the critical role of maternal health, nutrition, and prenatal care in preventing LBW. Interventions that address maternal education, nutritional improvement, regular antenatal visits, and timely management of maternal illnesses could play a pivotal role in reducing the incidence of LBW, ultimately improving neonatal outcomes.
